# Detailed analysis of putative genes encoding small proteins in legume genomes

**DOI:** 10.3389/fpls.2013.00208

**Published:** 2013-06-20

**Authors:** Gabriel Guillén, Claudia Díaz-Camino, Carlos A. Loyola-Torres, Rosaura Aparicio-Fabre, Alejandrina Hernández-López, Mauricio Díaz-Sánchez, Federico Sanchez

**Affiliations:** Departamento de Biología Molecular de Plantas, Instituto de Biotecnología, Universidad Nacional Autónoma de MéxicoCuernavaca, Morelos, México

**Keywords:** gene annotation, legume genomes, short open reading frames

## Abstract

Diverse plant genome sequencing projects coupled with powerful bioinformatics tools have facilitated massive data analysis to construct specialized databases classified according to cellular function. However, there are still a considerable number of genes encoding proteins whose function has not yet been characterized. Included in this category are small proteins (SPs, 30–150 amino acids) encoded by short open reading frames (sORFs). SPs play important roles in plant physiology, growth, and development. Unfortunately, protocols focused on the genome-wide identification and characterization of sORFs are scarce or remain poorly implemented. As a result, these genes are underrepresented in many genome annotations. In this work, we exploited publicly available genome sequences of *Phaseolus vulgaris*, *Medicago truncatula*, *Glycine max*, and *Lotus japonicus* to analyze the abundance of annotated SPs in plant legumes. Our strategy to uncover *bona fide* sORFs at the genome level was centered in bioinformatics analysis of characteristics such as evidence of expression (transcription), presence of known protein regions or domains, and identification of orthologous genes in the genomes explored. We collected 6170, 10,461, 30,521, and 23,599 putative sORFs from *P. vulgaris*, *G. max*, *M. truncatula*, and *L. japonicus* genomes, respectively. Expressed sequence tags (ESTs) available in the DFCI Gene Index database provided evidence that ~one-third of the predicted legume sORFs are expressed. Most potential SPs have a counterpart in a different plant species and counterpart regions or domains in larger proteins. Potential functional sORFs were also classified according to a reduced set of GO categories, and the expression of 13 of them during *P. vulgaris* nodule ontogeny was confirmed by qPCR. This analysis provides a collection of sORFs that potentially encode for meaningful SPs, and offers the possibility of their further functional evaluation.

## Introduction

Legumes comprise one of the largest plant families in the world, mainly as a result of the ability of its members to establish mutually beneficial root symbioses with soil bacteria and fungi that provide the plants with nutrients that are scarce in many soils. Leguminosae are second only to the Gramineae with respect to agricultural production and human and animal consumption (Udvardi, 2002; Graham and Vance, 2003).

The societal relevance of legumes has motivated considerable investment in legume genomics research in recent years. Although most resources have primarily focused on the development of genomic tools and biological investigation of the model legumes barrel medic (*Medicago truncatula*) and birdsfoot trefoil (*Lotus japonicus*), over the past years additional efforts have allowed the advance of soybean (*Glycine max*) and common bean (*Phaseolus vulgaris*) genomics (Gepts et al., 2005). Virtually complete genome sequences of *G. max*, *M. truncatula*, and *L. japonicus* were published in 2009 (Cannon et al., 2009), and the *P. vulgaris* genome is forthcoming. Considering that the final goal of legume genomics is to understand the organization and function of a unified legume genome in all its diversity, in this study we designed a strategy to uncover the neglected sector of potential genes encoding small proteins (SPs) in *G. max*, *L. japonicus, M. truncatula*, and *P. vulgaris* genomes.

Short open reading frames (sORF) are translated into SPs of 30–150 amino acids (aa) that play essential roles in eukaryotes (Kastenmayer et al., 2006; Kondo et al., 2010; Hanada et al., 2013). In plants, SPs are involved in a variety of processes, e.g., modulation of cell division and differentiation (Fletcher et al., 1999; Mortier et al., 2012; Hanada et al., 2013), stabilization, assembly, and dimerization of the PSII complex (Shi and Schröder, 2004), priming plant defenses (Silverstein et al., 2007; Gleason et al., 2008; Jung et al., 2009; Van de Velde et al., 2010), and regulating flowering time (Notaguchi et al., 2008). These examples illustrate that SPs are ubiquitous and function in plant physiology, growth and development. However, the identification and characterization of many other SPs remain largely unexplored.

Whole-genome tiling array studies (Stolc et al., 2005) revealed that sORFs have been underestimated in plants at a genome-wide level. In practice, sORFs are generally eliminated during genome annotation due to the arbitrary minimum open reading frame (ORF) cutoff of 100–150 aa used to reduce the likelihood of falsely categorizing protein-coding (mRNA) and non-coding (ncRNA) RNA (Dinger et al., 2008). Hence, the development of effective methods to recognize potentially functional sORFs is critical.

In this work, we collected all predicted ORFs coding for proteins in the available genome sequences of *P. vulgaris*, *M. truncatula*, *G. max*, and *L. japonicus*. We analyzed annotated sORFs equal to or smaller than 120 aa in length in each legume genome. For evidence of functionality, we compared each potential sORF by sequence similarity against the EST Gene Index database. Legume sORFs potentially encoding non-coding RNAs (ncRNAs) were predicted by the Infernal program (Nawrocki et al., 2009) and eliminated from our sORFs collections. Sequence similarity of annotated SPs to larger proteins in the genome was evaluated as well as their evolutionary conservation within plants. Additionally, potential functional sORFs were classified according to Gene Ontology (GO) (McCarthy et al., 2006). Finally, a time-course study of a group of selected sORFs was carried out during *P. vulgaris* nodule ontogeny by qPCR. This study demonstrates that is possible to identify functional sORFs in legume plant genomes, even in cases where the genome annotation is not yet complete.

## Materials and methods

### Strategy for large-scale discovery of sORFs in legume genomes

ORFs of *P. vulgaris*, *M. truncatula* (Young et al., 2011), *G. max* (Schmutz et al., 2010) and *L. japonicus* (Sato et al., 2008) were collected. For comparison with non-legume plants, ORFs from *Arabidopsis thaliana* (Swarbreck et al., 2008) and maize (*Zea mays*) (Schnable et al., 2009) were also gathered. Plant genome databases utilized for this were Phytozome (http://www.phytozome.com, Goodstein et al., 2012) and PlantGDB (http://www.plantgdb.org/*LjGDB*, Duvick et al., 2008). To calculate protein length frequencies, annotated proteins were downloaded in FASTA format. Protein lengths were summed within 40-aa bins. Amino acid bins were plotted as a function of protein size. ORF prediction was further confirmed by comparing non-coding regions (1 kb) immediately downstream from stop codons against the A. thaliana proteome by BLASTX. The average protein length for the four legume species occurs around 120 aa, therefore sORF candidate sets encoding proteins with a length equal to or less than 120 aa were compiled. Considering that not all putative ORFs will be of functional significance, we focused on properties that could be assessed using bioinformatics tools. Using the rationale that a functional gene should be transcribed, we compared each potential sORF by sequence similarity against the DFCI Gene Index database (http://compbio.dfci.harvard.edu/tgi/plant.html, Quackenbush et al., 2001; Tsai et al., 2005). BLASTN searches were conducted using default parameters (Altschul et al., 1990). Only those with an expectation value cutoff of 10 (*e*-^10^) were considered as positive matches. Potentially, ncRNAs were identified in the sORF candidate set by using the Infernal program (Nawrocki et al., 2009). To find common protein domains within each legume, predicted SPs were compared to larger proteins (longer than 120 aa) in the genome. Based on the supposition that potential sORFs are more likely to represent “true” genes if an ortholog can be found in another plant genome, we also evaluated the presence of each sORF in the *P. vulgaris*, *M. truncatula*, *G. max, L. japonicus, A. thaliana*, and *Z. mays* genomes (BLASTP, *e*-^10^). Finally, legume sORFs were classified on the basis of GO annotation (http://www.agbase.msstate.edu/cgi-bin/tools/GOanna.cgi, McCarthy et al., 2006), and the expression of 13 selected sORFs was tested during *P. vulgaris* nodule ontogeny by qPCR.

### Plant material, RNA extraction, and qRT-PCR analysis of expression

*Phaseolus vulgaris* L. cv. Negro Jamapa seeds were surface-sterilized in 10% (v/v) commercial sodium hypochlorite, rinsed with sterile water and germinated in the dark for three days on two layers of filter paper saturated sterile water at 28°C. Seedlings were inoculated with *Rhizobium tropici* CIAT899 (Martínez-Romero et al., 1991), transferred to vermiculite and grown in the greenhouse. Nodules and nodule-stripped roots were harvested at the indicated times, immediately frozen in liquid nitrogen, and stored at −70°C until use. Total RNA was isolated using the Fermentas GeneJET™ RNA purification kit according to the manufacturer's instructions (www.thermoscientificbio.com/fermentas/). RNA quantity was measured spectrophotometrically, and only the RNA samples with a 260/280 ratio between 1.9 and 2.1 and a 260/230 ratio greater than 2.0 were used for the analysis. The integrity of RNA samples was confirmed by agarose gel electrophoresis. For reverse transcription, 3 μg total RNA was treated with DNaseI (Fermentas), and 1 μg total RNA was reverse transcribed using the RevertAid™ H Minus First-strand cDNA synthesis kit (Fermentas) with anchored-oligo (dT) 18 primer according to manufacturer's instructions. For qPCR, 15 μl qPCR reactions using Maxima SYBR® Green qPCR Master Mix (Fermentas) were performed on an iCycle iQ5 apparatus (BioRad, www.bio-rad.com). The cycling conditions were: preheating for 5 min at 95°C followed by 30 cycles (denaturing for 15 s at 95°C, annealing and elongation for 15 s at 57°C and data acquisition at 81°C). A negative control reaction without template was also included for each primer combination. The melting curve protocol began immediately after amplification and consisted of 1 min at 55°C followed by 80 10 s steps with a 0.5°C increase in temperature at each step. The relative numbers for Ct of each gene (Table 1) were normalized to the house keeping gene Elongation factor 1-alpha (*Ef1*-α, Nawrocki et al., 2005). Data was analyzed using iQ™ 5 Optical System Software version 2.1 (BioRad). Three biological replicates were pooled and analyzed. At least six replicate PCR amplifications were performed for each sample.

**Table 1:** **List of oligonucleotides used in this work**.

Target^*^	**Forward sequence**	**Reverse sequence**
Phvul.008G217000	GTA CTT TCA GGG ACA TCA AAT GCA TC	GAG CAA ATT AGA AGC CGG AAC AGG
Phvul.008G217100	GTG GGT GAC GCC AAA TTC CTC G	GCA ATT GGC GTC GAA TCC ATA TGT AG
Phvul.002G030000	CGT GTG GTG TGT GCT CTG CTC T	GAA TCC TCT GTT GAA TCC CTC TGG
Phvul.002G127700	GGA GGA CTT TGA GGA GTA TGC TAA C	TTC AAT ATT CCA GGA CGG GAG GTG
Phvul.002G296000	GGC AGG TGT TAG CAA GAA TTC GAT G	CTA TCC CTT GAT CAA GAG ACG ACC
Phvul.006G001200	CTT ATC CTC CAC CTC CAC CTG TT	GCA TCC AAA ACA CAG CAG CAA CAC
Phvul.009G108100	CAA AGT TCA AGG AGG AGG CCA C	GAG TGT AAC CTT CAT GCA GGT GC
Phvul.010G012200	GTG TGG GTG TGG AAG CAG CTG	CCT TCA AAT TGG CCC TTC GCA G
Phvul.001G249700	CTT CCA TTG GAG CAC GTT CAG CT	CCC ACA CTT GAA CTT GTC ACC TTT C
Phvul.007G214100	GTG GTA GGA TTG CCC ATG CTA C	CAA GTA AAT CGT AGA AGG TCC TGA CT
Phvul.006G116900	GGC ATA CCG TAT GAG GAA ACC CT	GAG TTA TAC CTG TTC CGA TCG CC
Phvul.002G252800	TGA GCG TGG CAT CAT ACT TCG G	ATG GAG AGC GAT CCA GAC ATG G
Phvul.008G154900	CCC TTC TCA TAA CAA TTC TAG AAG AGC G	CTC AAT AAA GGA ACA CTG TTG TTC ATT GCG

* *Locus name in version 1.0 of Phytozome*.

## Results

### Length distribution of protein sequences in the sORF sets of analyzed legumes

**Figure 1:**
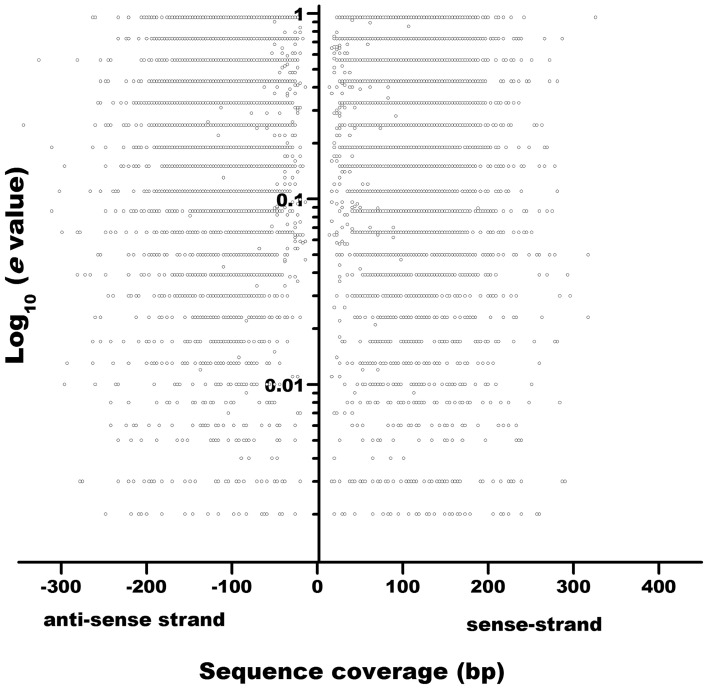
**Sequence similarity of 3′-non-coding sequences of putative *P. vulgaris* sORFs to the *Arabidopsis thaliana* protein collection.** 1 kb non-coding sequences (sense- and anti-sense strands) downstream stop codons of putative *P. vulgaris* sORFs are plotted as a function of similarity to *A. thaliana* proteins (*e*-^10^ value).

We collected 31,578, 55,715, 53,424, and 42,399 ORFs of *P. vulgaris*, *G. max, M. truncatula*, and *L. japonicus* genomes, respectively, and 27,414 (*A. thaliana*) and 63,540 (*Z. mays*) ORFs from non-legume plant genomes [http://www.phytozome.com (Goodstein et al., 2012) and PlantGDB (http://www.plantgdb.org/, Duvick et al., 2008)] (Tables 2, S1). Recent annotations of the *A. thaliana* genome include more sORFs relative to the earlier versions (Yang et al., 2011), indicating that the annotation of SPs is a key feature of improved annotations. Considering that a similar gene-calling procedure was followed to annotate all the legume genomes consulted in this work (Table 3), to evaluate the accuracy of this procedure, we retrieved 1 kb up- and downstream from each predicted ORF using the BioMart tool at the Phytozome website (http://www.phytozome.net/). We evaluated ORF prediction by comparing 1 kb of sequence downstream from the predicted stop codon of each putative *P. vulgaris* ORF against the A. thaliana proteome by BLASTX (Figure 1), an algorithm able to search a translated nucleotide sequence against a given protein sequence database. The lack of sequence similarity of these regions (represented by a low e value and low gene coverage) to known *A. thaliana* proteins indicated that most sORFs are not incorrectly annotated ORFs that are actually parts of longer ORFs.

**Table 2:** **Comparative values of genome size (in Mb), total open reading frames (ORFs) and short open reading frames (sORFs) encoding small proteins or peptides (SPs) in *Phaseolus vulgaris* (*P. vulgaris*), *Glycine max* (*G. max*), *Medicago truncatula* (*M. truncatula*), *Lotus japonicus* (*L. japonicus*), *Arabidopsis thaliana* (*A. thaliana*), and *Zea mays* (*Z. mays*)**.

	***P. vulgaris***	***G. max***	***M. truncatula***	***L. japonicus***	***A. thaliana***	***Z. mays***
Genome size	450–650	1115	550	470	157	2500
ORFs	31,578	55,715	53,424	42,399	27,416	63,540
sORFs	6170	10,461	30,521	23,599	6076	19,636

*Genome annotations and plant genome databases consulted are described in Materials and Methods*.

**Table 3:** **Analyzed plant genomes in version 9.0 of Phytozome**.

**Organism**	**Common name**	**Version**
*Arabidopsis thaliana*	Thale cress	TAIR version 10 (Swarbreck et al., 2008)
*Glycine max*	Soybean	US Department of Energy (DOE) Joint Genome Institute (JGI) Soybean (*Glycine max*) genome project version 1.1 (Schmutz et al., 2010)
*Medicago truncatula*	Barrel medic	Medicago Genome Sequence Consortium Mt3.5 version 4.0 (Young et al., 2011)
*Lotus japonicus*	Bird's-foot trefoil	Kazusa DNA Research Institute *Lotus japonicus* genome assembly build 2.5 (Sato et al., 2008)
*Phaseolus vulgaris*	Common bean	DOE-JGI Phaseolus genome project version 1.0
*Zea mays*	Maize	Unfiltered protein coding models from Maizesequence.org release 5b.60 (Schnable et al., 2009)

*The gene-calling procedure for each genome is described in detail in the indicated publication of the “Version” column*.

**Figure 2:**
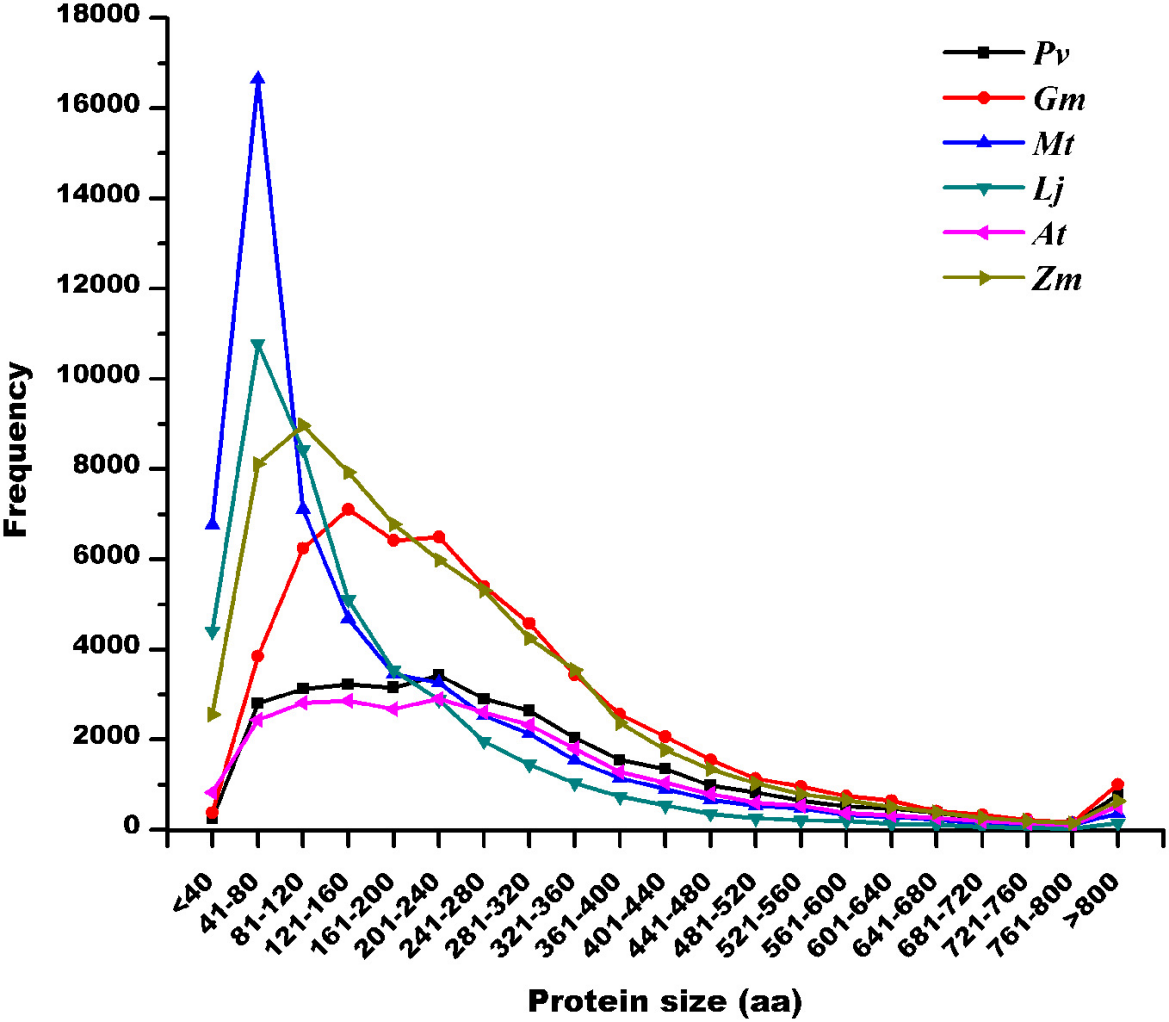
**Length distribution of predicted protein sequences in legume and non-legume plant genomes.** *Pv, Phaseolus vulgaris* protein sizes in *P. vulgaris* v0.9 annotation; *Gm, Glycine max* protein sizes in *G. max* v1.0 annotation; *Mt, M. truncatula* protein sizes in *Mt3.0* annotation; *Lj, L. japonicus* protein sizes in *Lj1.0* annotation; *At, Arabidopsis thaliana* protein sizes in genome release 9; and *Zm*, *Zea mays* protein sizes in Maize Golden Path B73 RefGen_v2.

Protein length distribution analysis (Figure 2) indicated that the highest frequency of predicted SPs was in the genomes of *M. truncatula* (57.2%) and *L. japonicus* (55.6%). By contrast, *P. vulgaris* (19.5%), *G. max* (18.8%), *A. thaliana* (22.2%), and *Z. mays* (30.9%) showed an abundance of SPs that was relatively homogeneous compared to the whole protein collection (Tables 2, S1, and Figure 2). Interestingly, protein abundance declined abruptly in all cases below 40 aa. This observation may suggest that in the cellular context, a minimum length is required to achieve a properly functioning protein.

### Evaluating sORF functionality by evidence of transcription

Compared to genomes, ESTs and other sources of transcript information are the most reliable evidence for gene expression and gene identification; introns and most pseudogenes are absent, the searched space is reduced compared to eukaryotic genes, and, typically, an mRNA encodes one protein (Frith et al., 2006). Unfortunately, the large volume of ESTs experimentally generated in each study (including a high number of short sequences) and the lack of functional annotation are frequent barriers for using ESTs for gene modeling and gene structure identification (Tsai et al., 2005).

**Figure 3:**
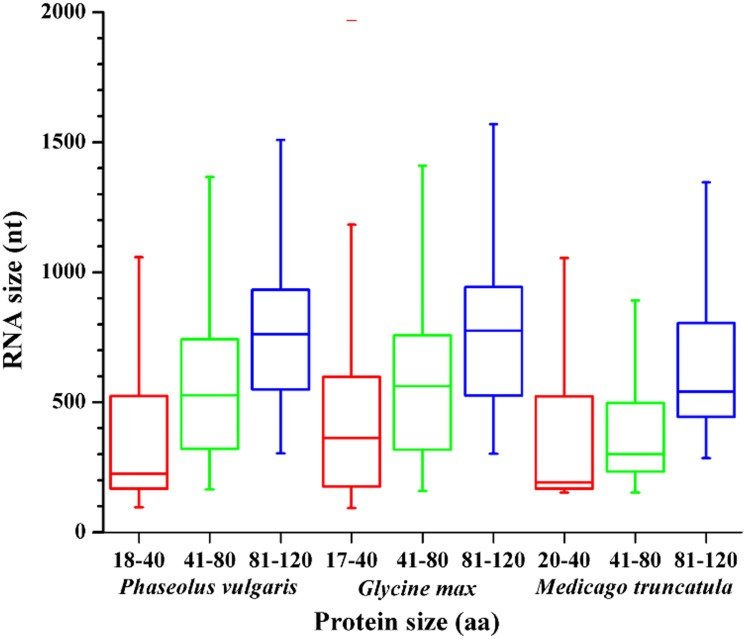
**RNA sizes for different ranges of protein size represented in a box and whisker plot.** The center lines indicate the medians, the top and bottom of each box indicate the first and third quartiles, and the whiskers extend to the most extreme data points.

To reduce the likelihood of falsely categorizing ncRNAs or transposable elements as mRNAs, many cDNA collections exclude transcripts under 500 nucleotides (nt) in length. Given that proteins of 120 aa or less may be encoded by transcripts of around 300 nt, this introduces a bias against evaluating the expression of short proteins. In this work, to assess whether sORFs were well represented in the EST libraries consulted (Tables 4, S1), we analyzed the lengths of ORFs that encode proteins of different sizes in legume species (Figure 3). For each protein size range (grouped in 40 aa bins) the RNA length varied between 180 and 800 base pairs, indicating that RNAs encoding potential SPs in those legumes would not be completely excluded from the publicly available EST libraries analyzed and that the databases could be used to evaluate sORF expression.

**Table 4:** **Evidence of transcription of legume sORFs based on ESTs**.

**Genome**	**sORFs**	**sORFs with expression evidence (ESTs)**	**EST source**
*P. vulgaris*	6170	2336	DFCI bean gene index release 4.0
*G. max*	10461	4665	DFCI soybean gene index, release 16.0
*M. truncatula*	30521	7687	DFCI medicago gene index, release 11.0
*L. japonicus*	23599	6744	DFCI L. japonicus gene index, release 6.0

*Each annotated sORFs was compared by sequence similarity against the DFCI Gene Index database (http://compbio.dfci.harvard.edu/tgi/plant.html, Quackenbush et al., 2001; Tsai et al., 2005). Only those with an expectation value cutoff of 10 (e^−10^) were considered as positive matches*.

Although ESTs are not sufficient to predict whether a gene is translated into a functional protein, their detection constitutes strong evidence of gene expression. BLASTN searches were conducted using default parameters (Altschul et al., 1990) and only those ESTs with an *e* value cutoff of 10 (*e*-^10^) were considered to be positive matches for any legume sORFs. We found 2334, 4665, 7687, and 6744 EST counterparts to sORFs predicted in *P. vulgaris*, *G. max*, *M. truncatula*, and *L. japonicus* genomes, which represent 37.82, 44.59, 25.18, and 28.57% of the total sORFs, respectively (Tables 4, S1).

A disadvantage of determining sORFs functionality exclusively based on transcript evidence is that these may be ncRNAs (Frith et al., 2006), which are difficult to distinguish from mRNAs encoding short proteins. ncRNA genes do not encode proteins but produce functional RNA molecules that play important biological functions in the cell. To determine whether candidate sORFs below 120 aa in length could be ncRNAs, an Rfam-based search with all legume sORFs using the Infernal program (Nawrocki et al., 2009) was performed. Just a proportion (10.5, 14.9, 0.06, and 5%, Table S2) of annotated as sORFs in the *P. vulgaris*, *G. max*, *L. japonicus*, and *M. truncatula* genomes were predicted as potential ncRNAs and eliminated.

### Comparison of common gene regions encoding putative SPs with genes encoding larger proteins, and evidence for orthologs

**Figure 4:**
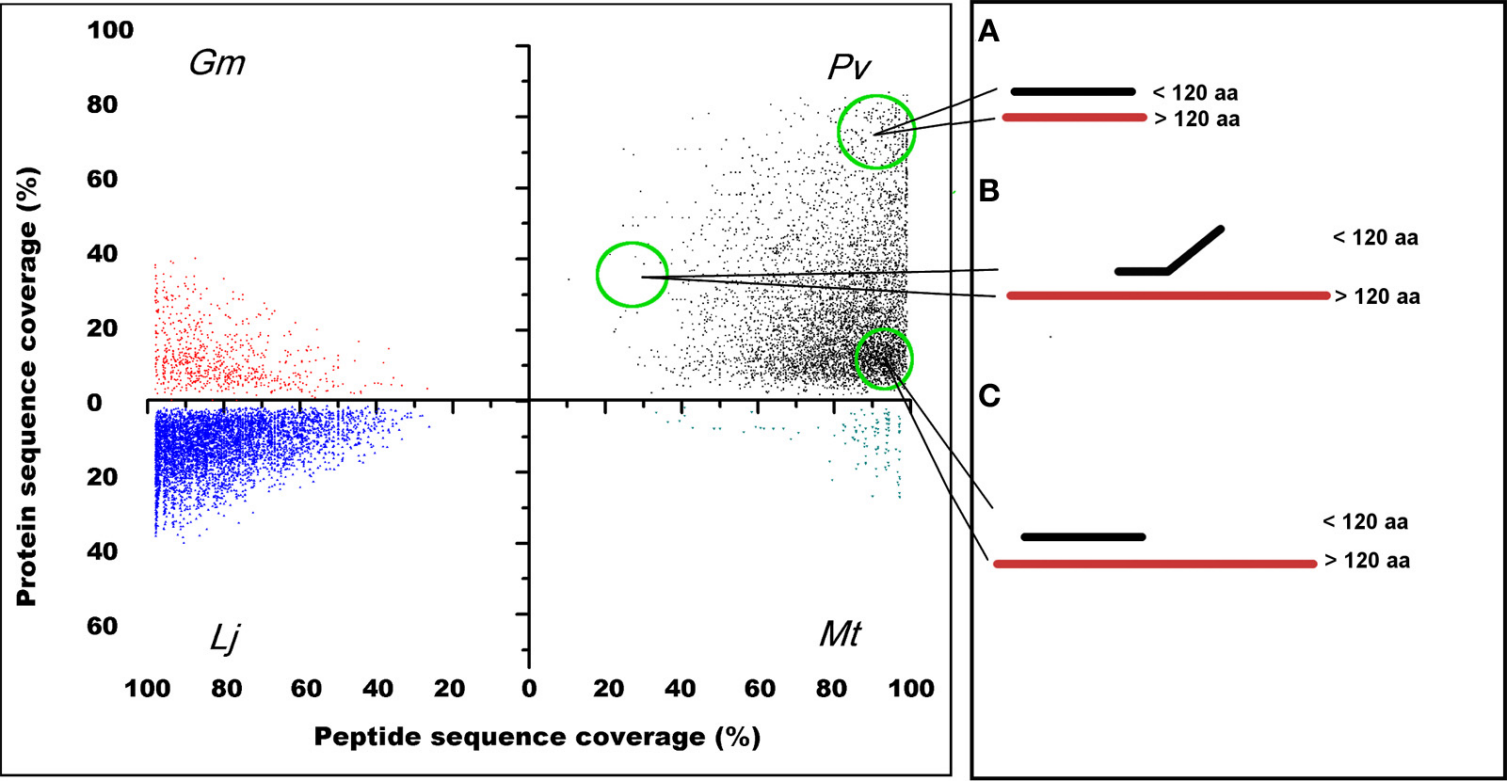
**Legume sORFs display common aa regions or domains with larger polypeptides of the same genome.** The identity level of *P. vulgaris*, *G. max*, *M. truncatula*, and *L. japonicus* predicted sORFs (peptide sequence coverage) is spread across several homologous proteins of variable size (protein sequence coverage) of the respective genome. As an example, **(A)** illustrates the distribution pattern of sORFs in *P. vulgaris* that are identical in sequence to other small proteins (slightly larger than 120 aa); in **(B)** sORFs that share a domain with larger polypeptides are included; and in **(C)** sORFs that are completely equivalent to regions or domains found in larger proteins are indicated.

Finding common regions or domains among proteins is a valid approach to distinguish protein-coding from non-coding genes (Frith et al., 2006; Kastenmayer et al., 2006). To test for sequence similarity between annotated SPs and larger proteins in the genomes of the legumes analyzed, we first compared *P. vulgaris*, *G. max*, *M. truncatula*, and *L. japonicus* SP to all other polypeptides (longer than 120 aa) included in the respective genome (Figure 4 and Table 5). With the exception of *M. truncatula* (2.4%), most annotated SPs in legumes share counterpart regions or domains with larger proteins Figure 4C, suggesting they may encode for functional SPs. Other potential SPs, particularly abundant in *P. vulgaris*, although identical in sequence, differ slightly in length among them Figure 4A or are partially related to other proteins Figure 4B. These groups could represent different members of protein families that have a common or related biological function (Marszalek et al., 1999; Schwaiger et al., 2003). Finally, there is a considerable number of SPs that lack similarity to other proteins in the same organism (Table 5, subsets <*e*^−5^ and “no hit”). This is particularly evident in *M. truncatula* and *L. japonicus*, where most sORFs (66 and 79%, respectively) are unique, and have no similarity to any longer proteins encoded in the genome. Although at least part of these sORFs may still encode genuine proteins, i.e., proteins that evolve at faster rates, in general these sORFs are listed as random ORFs arising in non-coding transcripts (Frith et al., 2006; Kastenmayer et al., 2006; Clamp et al., 2007). The *P. vulgaris* sORFs GC-content is similar to the average for the *P. vulgaris* genome (30–40%), which suggests that these sORFs may be actually protein-coding genes (Table S1 and Figure S1).

**Table 5:** **Frequency of potential sORFs sharing domains with larger polypeptides of the same genome**.

**Genome**	**No hit**	**<*e*^−5^**	**<*e*^−10^**	**>*e*^−10^**	**Total**
*P. vulgaris*	1200	3768	757	818	6170
*G. max*	6265	3265	340	591	10,461
*M. truncatula*	20,301	1972	195	53	30,521
*L. japonicus*	18,677	3697	694	531	23,599

*BLASTP analyses were performed with predicted SPs (<120 aa) against all other proteins (longer than 120 aa) in each legume genome. Obtained e values were used to cluster SPs according to similarity to other proteins*.

Since sORFs are more likely to represent functional proteins if an ortholog can be found, sORFs detected in each legume genome were compared to each other based on sequence similarity (BLASTP, *e*^−10^). We also included in this analysis sORFs from *A. thaliana* and *Z. mays* to assess which sORFs might have evolved from ancestral genes present in a common plant ancestor, but lost in other legumes. A large percentage of *P. vulgaris* and *G. max* SPs were found to have orthologs in model legumes, and also in other plants (Table 6). In contrast to *P. vulgaris* and *G. max*, only a small fraction of SPs from *M. truncatula* and *L. japonicus* (less than 10% in all comparisons) shared sORFs or had orthologs in non-legume plants (Table 6).

**Table 6:** **Number of sORFs from legume species predicted to be orthologous to each other**.

	***P. vulgaris***	***G. max***	***M. truncatula***	***L. japonicus***	***A. thaliana***	***Z. mays***
*P. vulgaris*		3586 (58.1%)	2345 (38.0%)	2444 (39.6%)	2038 (33.0%)	1616 (26.2%)
*G. max*			5414 (51.8%)	5391 (51.5%)	4438 (42.0%)	3796 (36.3%)
*M. truncatula*				2812 (9.2%)	1611 (5.3%)	1604 (5.2%)
*L. japonicus*					1535 (6.5%)	1906 (8.0%)
*A. thaliana*						1392 (22.9%)

*Total number of shared sORFs (BLASTP e^−10^) among legume or non-legume genome plants are indicated*.

### SP classification based on gene ontology

**Figure 5:**
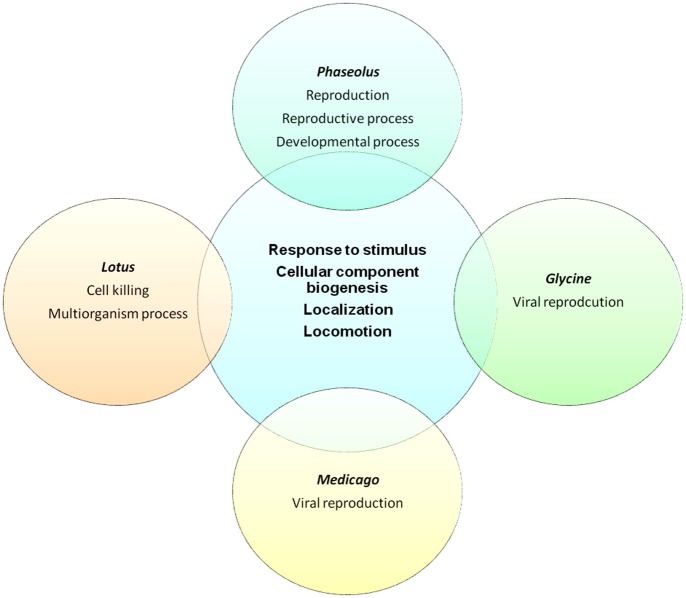
**Venn diagram representing the distribution of GO categories found in each legume genome.** Around 15% of all sORFs in legumes were included in “response to stimulus” and close to 20% were related to “localization” GO categories. The Fisher's exact test (Routledge, 1998) was applied to determine which GO categories were statistically over-represented compared to all proteins of the genome (*p* < 0.05, corrected by Benjamini adjustment).

One of the most important tools to establish ontologies is GO analysis (Ashburner et al., 2001), which depicts the potential function of a gene product in a cellular context; thus, annotation of putative SPs encoded by sORFs in legume genomes could provide valuable information to interpret their biological role. Out of 6170 potential sORFs in *P. vulgaris*, 4590 are homologous to *A. thaliana* proteins, and 2670 of them were associated with a GO “biological process.” The Fisher's exact test (Routledge, 1998) was applied to determine which GO categories were statistically over-represented compared to all proteins of the genome (*p* < 0.05, corrected by Benjamini adjustment). Interestingly, 14% of the total sORFs were preliminary classified into “response to stress” (Figure 5 and Table S3). A similar ratio was obtained after analyzing the SPs contained in the genomes of *G. max*, *M. truncatula*, and *L. japonicus* (Figure 5 and Table S3). Remarkably, only in *P. vulgaris* were a considerable percentage of sORFs (6.4%) grouped into “developmental process.”

### Confirming sORF functionality by analysis of transcript expression in *P. vulgaris*

**Figure 6:**
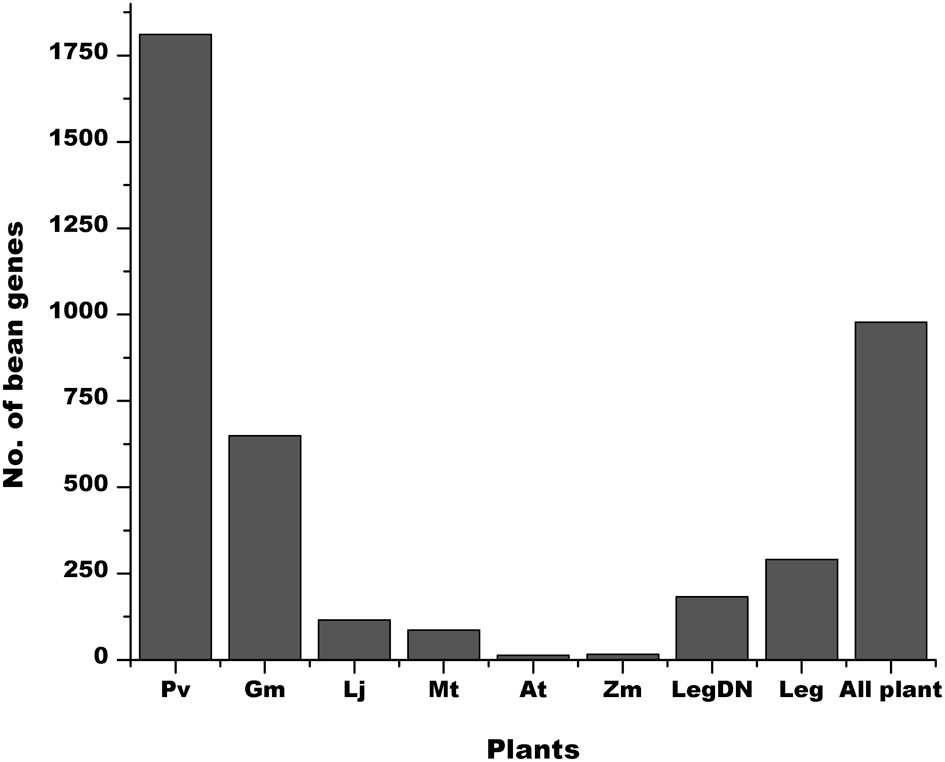
**Some sORFs in *P. vulgaris* are shared with other plants.** The graph shows the number of *P. vulgaris* sORFs exclusively found in this plant (Pv) compared to those that are also present in *G. max* (*Gm*), *M. truncatula* (*Mt*), *L. japonicus* (*Lj*), *A. thaliana* (*At*) and *Z. mays* (*Zm*). It also shows the number of sORFs of legumes that form determinate (LegDN) or undeterminate (Leg) nodules. The *All plants* bar represents the number of sORFs that are common to all plant species evaluated.

The *P. vulgaris* genome has 6170 annotated SPs equal or smaller than 120 aa (Tables 2, S1). A high proportion of these genes are exclusively present in the *P. vulgaris* genome (Figure 6, Pv), whereas others have a counterpart in other legume [*G. max* (*Gm*), *M. truncatula* (*Mt*) and *L. japonicus* (*Lj*)] or non-legume [*A. thaliana* (*At*), and *Z. mays* (*Zm*)] genomes (Figure 6). As expected, a higher proportion of the predicted SPs in *P. vulgaris* were also identified in legumes that form determinate nodules (Figure 6, LegDN), such as *G. max*, *L. japonicus*, and *Vignia unguiculata*. However, an important number of these sORFs are also found in legumes that form indeterminate nodules, such as *M. truncatula*, *Pisum sativum*, and *Trifolium repens* (Figure 6, Leg).

Based on ESTs, 2336 sORFs had evidence of gene expression (Tables 4, S1). By comparing the sORFs of *P. vulgaris* with larger proteins of the *P. vulgaris* genome and with sORFs of other legume (*G. max, M. truncatula*, and *L. japonicus*) and non-legume plants (*A. thaliana* and *Z. mays*) we determined that 5521 had counterpart regions or domains found in larger proteins of the *P. vulgaris* genome (Figure 4) and that 3914 contained a high level of identity (BLASTP *e*^−10^) to sORFs found in other plant species (Table 6). Many of the sORFs in *P. vulgaris* were detected by more than one approach (Figure 8). For example, a large number of sORFs were transcribed and contained common regions found in larger *P. vulgaris* proteins, or were transcribed and had potential orthologs in other legume or non-legume plants (Figures 6, 8). sORFs detected by all techniques were deemed likely to be *bona fide* genes.

**Figure 7:**
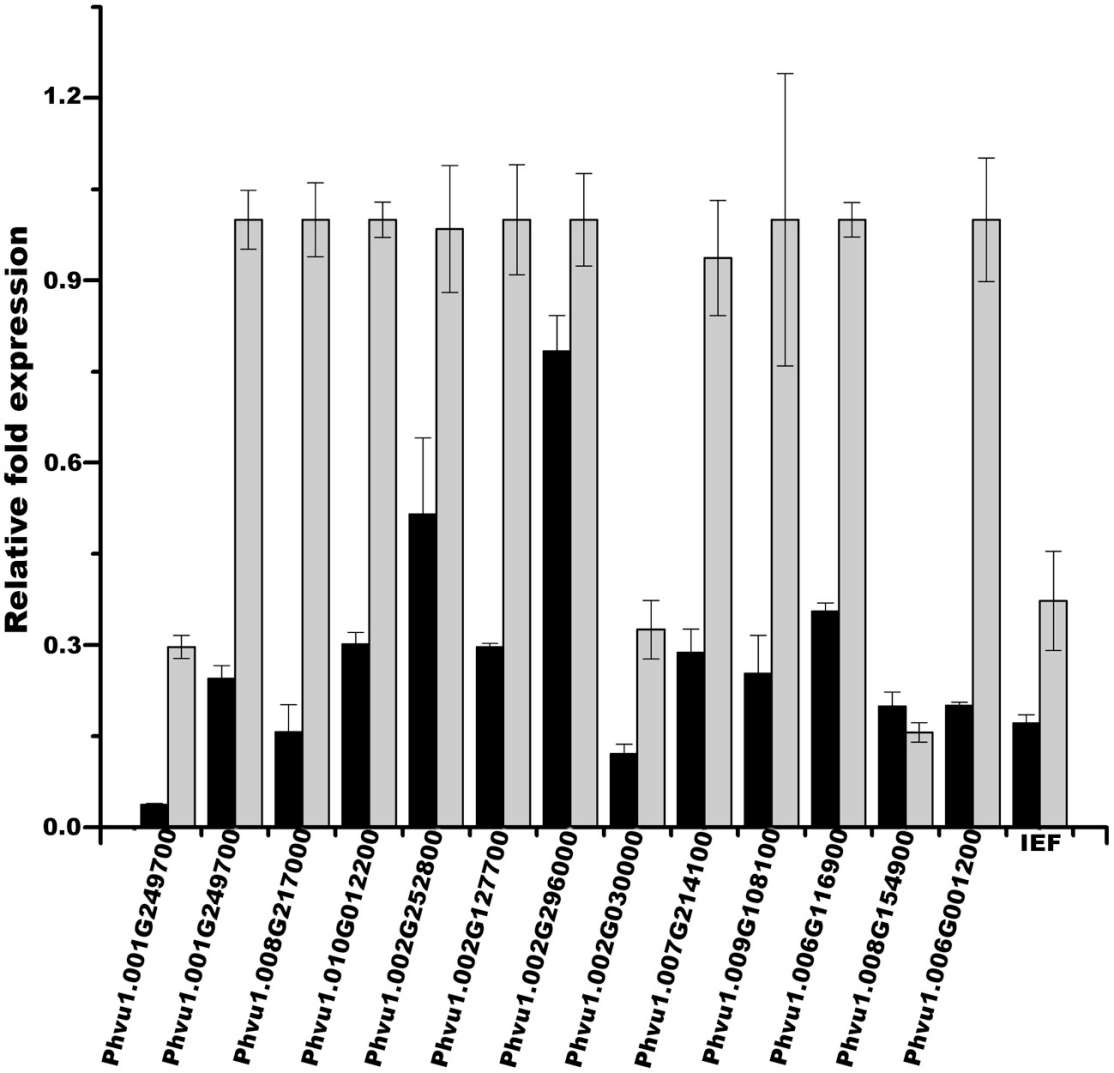
**sORFs expression during nodule ontogeny.** The gene expression of a small group of sORFs was confirmed by qPCR. Relative expression levels of a selected group of sORFs (Table 7) were determined in nodules and nodule-stripped roots at the indicated times by qPCR. Total RNA was isolated from each biological sample. First strand cDNA was synthesized and subjected to qPCR as described in Materials and Methods. Expression levels were normalized against Elongation factor 1-alpha (*Ef1*-α) values. Ratios of expression in nodule-stripped roots to nodules are graphed. These values represent the mean and SD of triplicate experiments.

**Figure 8:**
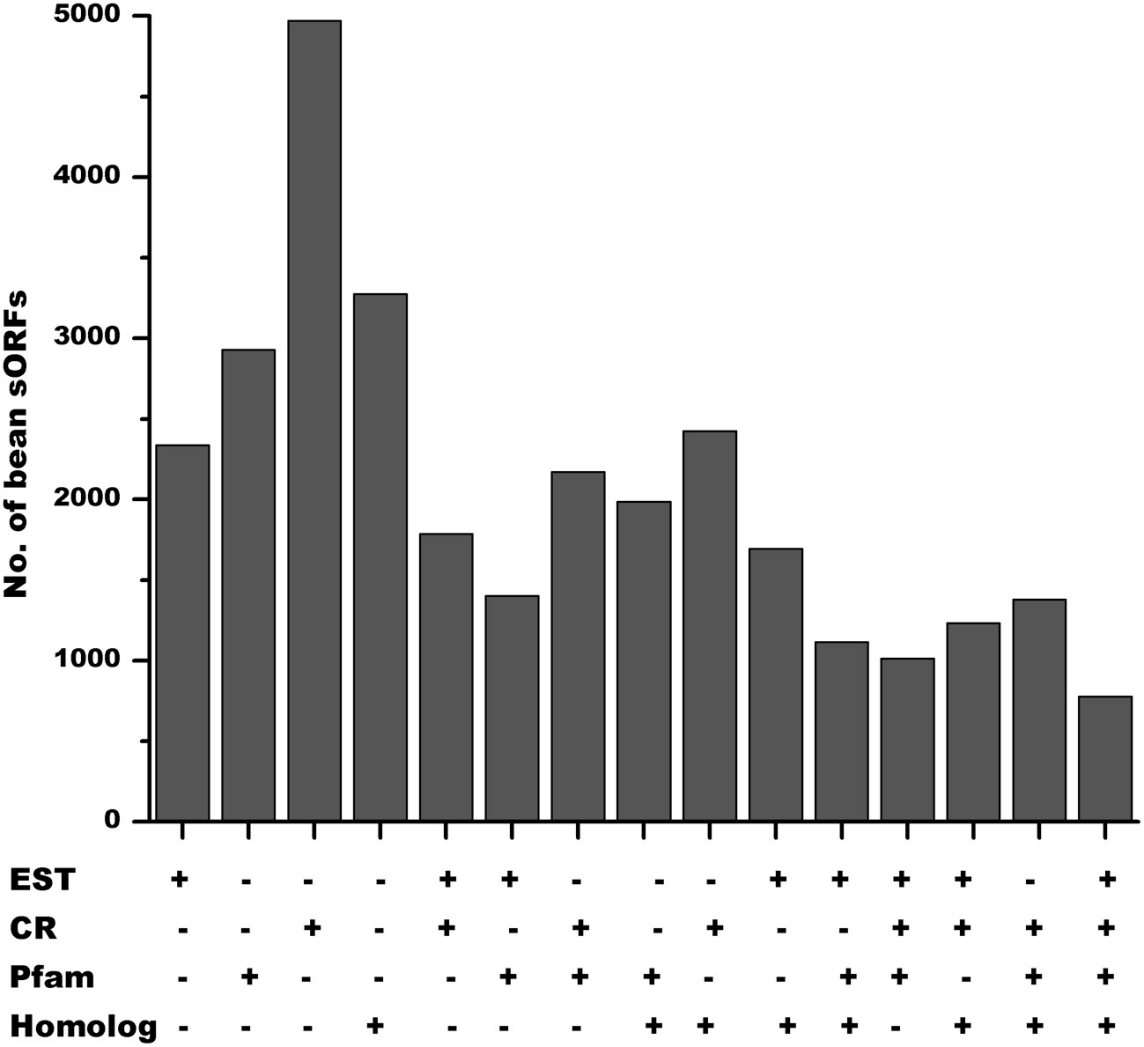
**Evidence of functional SPs in *P. vulgaris*.** Out of 6170 annotated sORFs in the genome of *P. vulgaris*, 2336 had expression evidence (DFCI Gene Index database), 2929 shared common regions or domains with other proteins (larger than 120 aa) of *P. vulgaris* and 3274 were homologous to SPs found in different plant species. According to the Phytozome annotation, 4970 belong to one or more protein families. 2553 sORFs in *P. vulgaris* have at least one of these types of evidence of functionality, whereas 2321 have two of them and a total of 776 sORFs have all of them.

To test the efficiency of our method in validating legume sORFs, we explored by qPCR the expression of 13 sORFs selected from a group consisting of 186 that are exclusively present in determinate nodules (Gene Index database, http://compbio.dfci.harvard.edu/tgi/plant.html, Quackenbush et al., 2001; Tsai et al., 2005; Figure 8, LegDN). *P. vulgaris* roots were inoculated with *Rhizobium tropici* CIAT899 (Martínez-Romero et al., 1991). Results from each nodule developmental stage were compared to age equivalent un-inoculated roots. With the sole exception of Phvul.008G217000, all other selected sORFs have more than one evidences of functionality (Table 7). Interestingly, all selected sORFs (even Phvul.008G217000) are expressed in *P. vulgaris* root nodules of 10 and 14 days after inoculation (d.p.i.) compared to age equivalent un-inoculated roots (Table 7 and Figure 7).

**Table 7:**
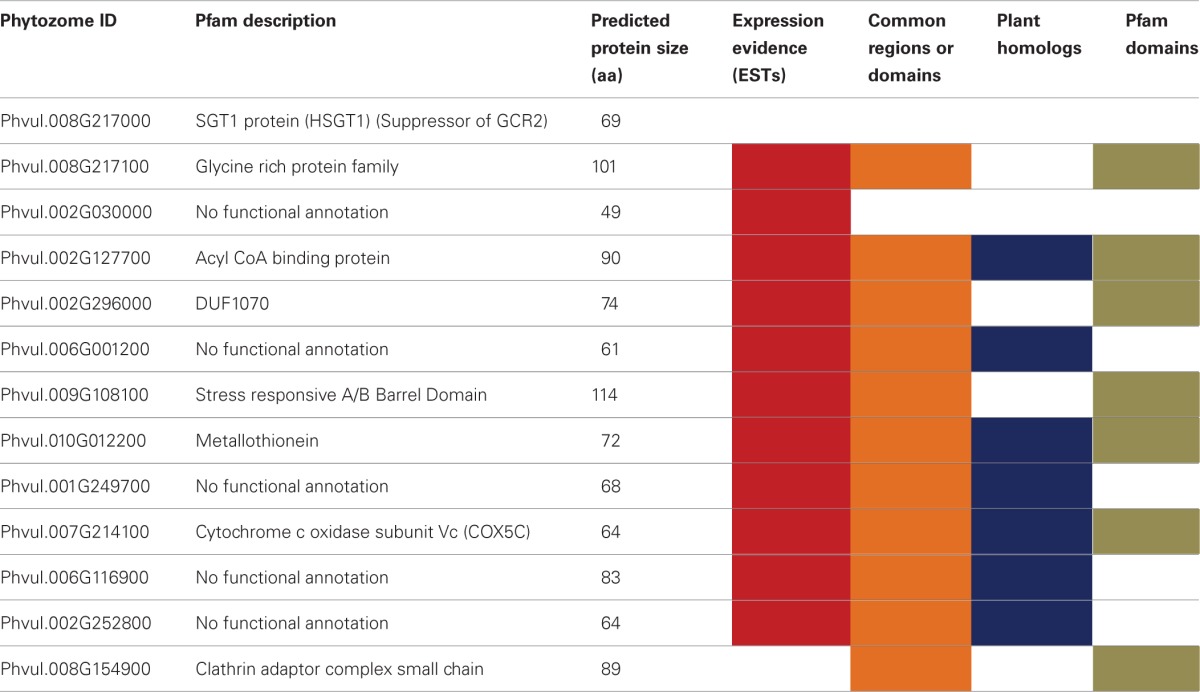
**sORF expression during nodule ontogeny**.

*The gene expression of a small group of sORFs was confirmed by qPCR (Figure 7). Other in silico evidence of their presumed functionality are also included in this table: EST matches (BLASTN e^−10^), common regions or domains shared with larger proteins of the P. vulgaris genome (Common regions or domains), evidence of homologs in other plant species, and identification of known protein domains (Pfam description). Colored boxes indicate positive evidence*.

## Discussion

The release of the *Arabidopsis thaliana* genome sequence in Arabidopsis Genome Initiative (2000) marked the beginning of the plant genomics era. In the last decade, diverse plant genome sequencing projects, including the Legume Crops Genome Initiative (Gepts et al., 2005), coupled with the development of powerful bioinformatics tools has facilitated massive data analysis. However, there are still a considerable number of proteins without assigned functions.

It has been reported that some SPs (30–150 aa in length) are involved in plant cell signaling and other processes in plants, but the overall scope of their abundance and biological relevance is still unknown. Here, sORFs encoding proteins of 120 aa or less in length in *P. vulgaris*, *G. max*, *M. truncatula*, and *L. japonicus* were analyzed and compared to those from two non-legume genomes (*A. thaliana* and *Z. mays*).

Our data indicate that the frequency distribution of potential SPs in the genomes of *P. vulgaris* and *G. max* are similar to that in *A. thaliana* (Figure 2), a vastly explored non-legume plant genome. Interestingly, the highest frequency of sORFs was found in the genomes of *M. truncatula* and *L. japonicus*, which are the two best-studied genomes in leguminous plants, and just a small proportion of these sORFs (0.06 and 5% in *L. japonicus* and *M. truncatula*, respectively) were identified as potential ncRNAs (Infernal program; Nawrocki et al., 2009). However, the existence of an ORF in genomic sequence does not necessarily demonstrate the existence of a functional gene.

We evaluated ORF prediction by comparing 1 kb of sequence downstream from the predicted stop codon of each putative *P. vulgaris* ORF against the *A. thaliana* proteome by BLASTX (Figure 1). In *P. vulgaris* genome, the average intron length is 400–500 bp and 75% of all introns are below 875 bp (data not shown). Therefore, comparing 1 kb downstream of the predicted stop codon against the protein database of *Arabidopsis thaliana*, (in the six possible reading frames), should be sufficient to reduce false positives created by truncated gene models. In other species, such as maize, that contains larger introns, a larger window would need to be analyzed to resolve this potential annotation issue.

As the first evidence for sORF functionality, we searched for evidence of expression in EST collections (Tables 4, S1). By this method, we estimated that between 25 and 45% of all potential SPs encoded in the genomes analyzed are represented by at least one EST (Tables 4, S1). These results imply that the majority of sORFs predicted in legume genomes are under-represented in the EST collections consulted, particularly those that encode for tiny proteins (less than 40 aa). Although unlikely, these data could indicate that some of the sORFs are simply random ORFs, rather than valid protein-coding genes.

Domains are the structural and functional building blocks of proteins. Given that most protein-encoding genes share conserved domains, we compared *P. vulgaris*, *G. max*, *M. truncatula*, or *L. japonicus* annotated SPs against longer polypeptides in their respective genomes (Figure 4 and Table 5). Most potential SPs in legumes were found to be equivalent to regions or domains found in larger proteins Figure 4C. Interestingly, the distribution pattern of a large number of SPs in *P. vulgaris* indicated a remarkable abundance of proteins that are identical in sequence but vary slightly in length Figure 4A. Domain length variations in proteins can result in functional differences such as in some actin-binding protein families, where domain length variations are related to their mechanical stability in binding F-actin (Marszalek et al., 1999; Schwaiger et al., 2003). Therefore, it is possible that these groups of sORFs share similar biological functions.

A considerable number of sORFs in all legumes analyzed showed “low-identity” (Table 5, subsets <*e*^−5^ and “no hit”). This was particularly evident among sORFs of *M. truncatula* and *L. japonicus*, where most (66 and 79%, respectively) were unique. Although it is unclear to what extent these sORFs encode real proteins, proteins that are both short and dissimilar to any known protein in the genome could be acquired by horizontal gene transfer or could represent novel genes that arose after divergence. Both possibilities should be evaluated in greater detail.

Most sORFs of *P. vulgaris* and *G. max* had orthologs in other legumes and plants (Table 6). In general, orthologs retain the same function through evolution (Tatusov et al., 1997); thus, sORFs found in non-legume plants are likely to be related to common biological and chemical processes in plants (Hanada et al., 2013), whereas sORF orthologs present uniquely in *Medicago* or *Lotus* may reflect a distinctive function of legumes, such as nodulation. Again, just a small fraction of sORFs from *M. truncatula* and *L. japonicus* (less than 10% in all comparisons) were shared or had orthologs in non-legume plants (Table 6). This fact, together with the absence of domains shared with larger polypeptides (Figure 4 and Table 5), could reflect speciation events leading to a variety of large gene families. Indeed, synteny comparisons between *Medicago* and *Lotus* indicate that a genome duplication event occurred after speciation (Cannon et al., 2006). Alternatively, these proteins could arise from ancestral genes present in a common ancestor, but lost in other legumes. An example of a remarkable family of proteins that share this feature is the nodule-specific cysteine-rich (NCR) legume peptides involved in regulating the differentiation of soil nitrogen-fixing bacteria during symbiosis (Van de Velde et al., 2010).

Leguminous plants are able to establish symbiotic relationships with soil nitrogen-fixing bacteria (commonly called rhizobia), an association that leads to the formation of a new organ in the plant, the symbiotic nodule. Nodulation in legumes provides a major conduit of available nitrogen into the biosphere; therefore, its study is of great importance in sustainable agriculture. We are particularly interested in studying diverse signaling mechanisms during the organogenesis of nitrogen-fixing nodules in *P. vulgaris*. For this reason, we explored the expression of sORFs that are exclusively present in determinate nodules (Figure 6). We selected 13 sORFs whose expression was related to nodulation, i.e., for which we found evidence of expression only in EST libraries generated from modulated plants (Gene Index database, http://compbio.dfci.harvard.edu/tgi/plant.html, Quackenbush et al., 2001; Tsai et al., 2005). All of these sORFs were homologous to other larger *P. vulgaris* proteins and some of them had potential orthologs in other plant species. As expected, all tested sORFs were expressed during nodule ontogeny (Table 7 and Figure 7).

The identification of novel genes is an urgent requirement for gene investigation in the age of genomics. The strategy for discovery of potential sORFs at a large-scale in legume genomes described here will contribute to their annotation and identifies new potential regulators of diverse biological processes in plants that should improve our understanding of plant biology. Our analysis revealed that in *P. vulgaris*, 2336 potential sORFs are transcribed, 2929 potential SPs sharing common regions or domains with other proteins of *P. vulgaris* and 3274 were homologous to other SPs found in different plant species (Figure 8). Remarkably, 2553 putative SPs in *P. vulgaris* have at least one evidence of functionality, 2321 have two of them and a total of 776 sORFs have all of them. sORFs detected by all techniques are likely to be *bona fide* protein coding genes.

Caveat to this approach: SPs below 40 aa in length, those that are encoded by genes with low expression, SPs that fall into protein families that are entirely species-specific or that contain unknown protein domains are not here represented. However, the functionality of sORFs could also be validated by highly sensitive methods to detect gene expression, such as qPCR, LC-MS, or HPLC-MS in a particular tissue or cell compartment (Wienkoop and Saalbach, 2003), in a wide range of growth stages or stress conditions (Zhang et al., 2006; Yang et al., 2011), and by gain-of- and loss-of-function (Hanada et al., 2013) approaches.

### Conflict of interest statement

The authors declare that the research was conducted in the absence of any commercial or financial relationships that could be construed as a potential conflict of interest.

## Supplementary material

Table S1 **List of sORFs in *P. vulgaris* and evidences of functionality**.

Table S2 **Infernal data**.

Table S3 **Venn diagram data**.

Figure S1 **GC-content of promoters, ORFs and sORFs in *P. vulgaris***.
