# Supplementary figures and images for: Detailed analysis of putative genes encoding small proteins in legume genomes
Source: Front Plant Sci. 2013 Jun 20;4:208. doi: 10.3389/fpls.2013.00208 (PMC3687714; doi:10.3389/fpls.2013.00208)

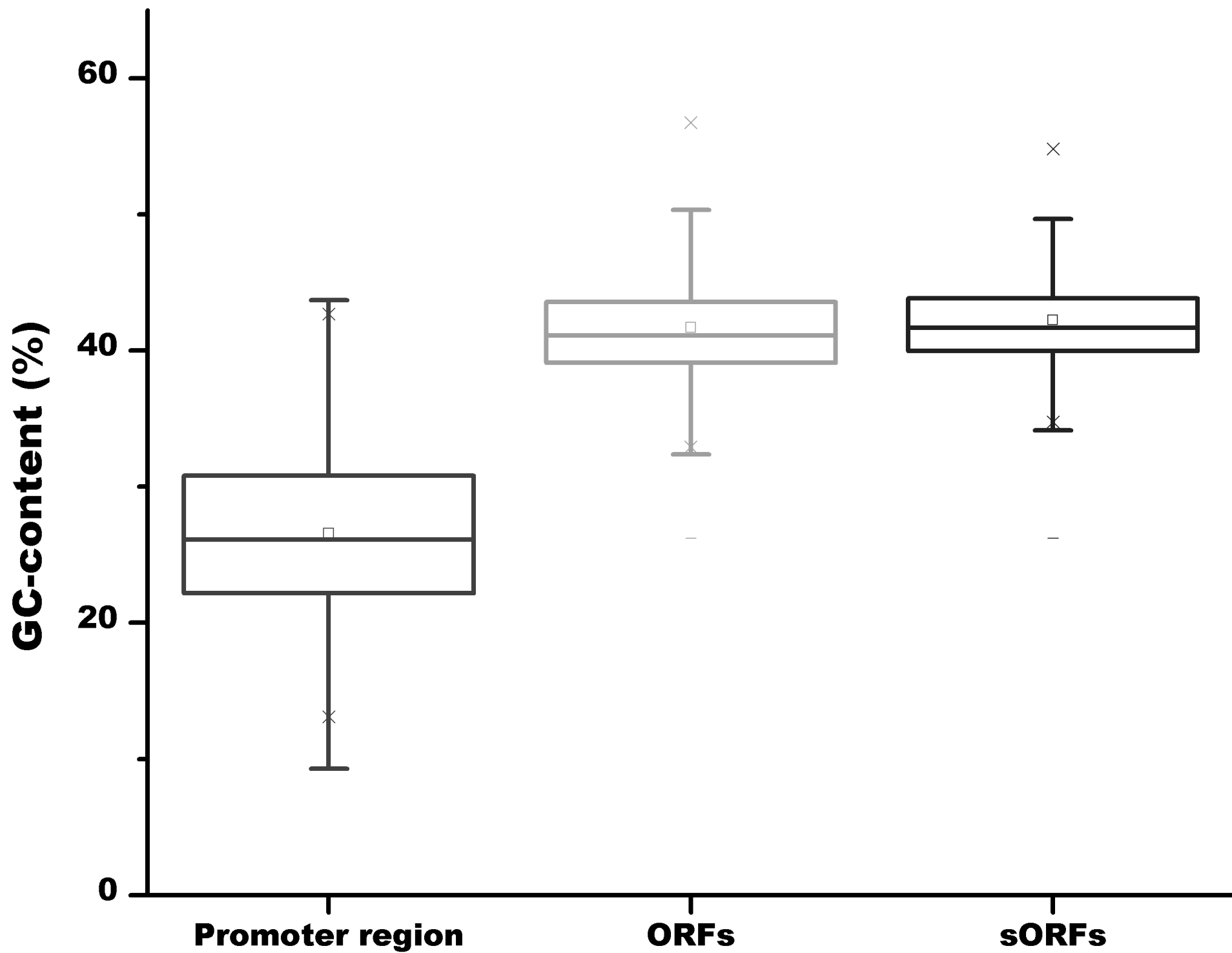

Supplement: Figure S1 — GC-content of promoters, ORFs and sORFs in P. vulgaris. [file Presentation1.PDF]
